# A New Candidate as a Hemostatic Agent for Difficult Situations During Variceal Bleeding: Ankaferd Blood Stopper

**DOI:** 10.4103/1319-3767.77248

**Published:** 2011

**Authors:** Ersan Ozaslan, Tugrul Purnak, Ayla Yildiz, Ibrahim C. Haznedaroglu

**Affiliations:** Department of Gastroenterology, Numune Education and Research Hospital, Ankara, Turkey; 1 Department of Hematology, Hacettepe Faculty of Medicine, Ankara, Turkey

**Keywords:** Ankaferd blood stopper, duodenal varices, endoscopy, gastric varices

## Abstract

Variceal bleeding is the most challenging emergent situation among the causes of upper gastrointestinal bleeding. Despite substantial improvement, a need remains for therapeutic armamentarium of such cases, which is easy, effective and without side-effect. Ankaferd blood stopper (ABS) is a standardized herbal extract acting as a hemostatic agent on the bleeding or injured areas. In this observational study, a total of four patients with variceal bleeding were treated with endoscopic ABS application. The lesions were bleeding gastric varices (n:3) and bleeding duodenal varix (n:1). ABS was selected as a bridge to definitive therapies due to unavailability or inappropriateness of bleeding lesions to conventional measures. ABS was instilled or flushed onto the bleeding areas by sclerotherotherapy needle or heater probe catheter. Periprocedural control of the bleeding was achieved in all instances. Thereafter, on an elective basis, two patients with gastric varices underwent cyanoacrylate injection, while third underwent Transjugular intrahepatic portosystemic shunt and embolization. The patient with duodenal varix refused further therapy, after a few hours after admission and was discharged. He again presented the same day with rebleeding, but died before any attempt could be made to control his bleeding. ABS seems to be effective in cases of variceal bleeding as a bridge to therapy. Its major advantages are the ease of use and lack of side-effects.

Endoscopic variceal ligation and/or injection sclerotherapy are well-established measures of bleeding esophageal varices despite some failures and complications; hovewer, the management of gastric and ectopic varices is more challenging. Cyanoacrylate injection is currently recommended for endoscopic treatment of gastric variceal bleeding.[1] Transjugular intrahepatic portosystemic shunt (TIPS) is recommended for patients in whom bleeding recurs despite combined medical and endoscopic therapy. When the standard treatment methods are inefficient or unavailable, it is obvious that the simplier methods should be available until TIPS procedure can be performed in a well-equipped center. In addition to controversies about the technical details and potential adverse complications of cyanoacrylate, such as thromboembolism,[2] it is costly and largely unavailable in many centers. In Turkey, cyanoacrylate is not reimbursable, and even direct purchase by patients may result in delays to therapy in urgent settings.

Ankaferd blood stopper (ABS) is a Turkish folkloric herbal extract obtained from five different plants, *Thymus vulgaris* (thyme), *Glycyrrhiza glabra* (licorice), *Vitis vinifera* (grape), *Alpinia officinarum* (lesser galangal) and *Urtica dioica* (stinging nettle). The use of topical ABS as a hemostatic agent has been approved by the Turkish Ministry of Health for the management of dermal, external postsurgical and postdental surgery bleedings.[3] The *in vivo* hemostatic effect of ABS was evaluated in rats pretreated with acetylsalicylic acid or enoxaparine as well as in a swine model.[4,5] Recently, a prospective, controlled clinical trial (phase III) of ABS in bleeding during tonsillectomy has been reported.[6] The successful usage of ABS has been described in various gastrointestinal (GI) scenarios as case reports, such as Dieulafoy lesion[7] and neoplastic GI bleeding.[8] Moreover, bleeding due to tooth-extraction in a cirrhotic patient with deficient hemostasis was controlled by ABS.[9]

## CASE REPORTS

This is an observational study of four patients presenting with variceal bleeding. ABS was used in five bleeding sessions (twice in one patient) as a primary hemostatic agent due to unavailabilities or difficulties of the conventional measures. We selected ABS as a hemostatic agent in gastric varices cases (patients 1-3) due to unavailability of cyanoacrylate and in patient 4 due to huge size of duodenal varices. Moreover, massive bleeding and hemodynamic instability of cases forced us to apply a more practical measure. Patients demographics, definition of bleeding and follow-up during and after endoscopic procedures were noted [Tables 1 and 2].

**Table 1:** The characteristics of patients and initial bleeding

Patient features			Definition of bleeding		
Age/Sex	Diagnosis	Bleeding source	Symptoms and signs	Hb /Hct	Transfusion*
40/F	Cryptogenic cirrhosis	Gastric varices (IGV-1, large, tortuous)	Hematemesis Hypotension (80/50 mmHg) Tachycardia (135/min)	6.9 gm/dL (19.2%)	3 units whole blood
65/F	Cryptogenic cirrhosis	Gastric varices (IGV-1, large, tortuous)	Hematemesis Hematochezia Hypotension (88/55 mmHg) Tachycardia (1115/min)	8.1 gm/dL (24%)	3 units whole blood
66/M	Cryptogenic cirrhosis	Gastric varices (with small esophageal varices, large, tortuous)	Hematemesis Melena Hypotension (85/55 mmHg) Tachycardia (122/min)	8.0 gm/dL (22%)	2 units whole blood
48/M	Alcoholic cirrhosis	Duodenal varices (huge, tortuous)	Hematemesis Melena Hypotension (95/60 mmHg) Tachycardia (111/min)	8.8 gm/dL (26%)	2 units RBC

* Total number of transfusions performed before and during the endoscopy. IGV-1: isolated gastric varices

**Table 2:** The details of procedures and follow-up

Endoscopic features			Postendoscopic follow–up	
No.	Reason of ABS use	Procedure/ the time of hemostasis achieved	Early	Late
1	Massive bleeding Hemodynamic instability Unavailability of cyanoacrylate	ABS (20 ml) 8^th^ minute	Elective cyanoacrylate injection on 3^rd^ day	Stable at 7^th^ mo
2	Massive bleeding Hemodynamic instability Unavailability of cyanoacrylate	ABS (15 ml) 5^th^ minute	Elective cyanoacrylate injection on 3^rd^ day, spurting during the procedure, restopped by ABS (25 ml)	Stable at 5^th^ mo
3	Massive bleeding Hemodynamic instability Unavailability of cyanoacrylate	ABS (15 ml) 5^th^ minute	No injection of cyanoacrylate Elective TIPS and embolization with coils at 7^th^ day	Stable at 3^rd^ mo
4	Recent bleeding Unavailability of cyanoacrylate	ABS (7 ml) 3^rd^ minute	Voluntarily discharge from hospital after six hours of observation Exitus from rebleeding sixteen hours after the procedure	

ABS: endoscopic topical use of Ankaferd blood stopper; GV: Gastric varices ; mo: Months

For all patients, written informed consent regarding the off-label use of ABS as a means of attaining hemostasis had been obtained from themselves or relatives. In all procedures, ABS was applied topically at varying doses [Table 2]. Fujinon^®^(EG450WR5, Fujionon Corporation, Saitama, Japan) diagnostic endoscopes were used for the topical application of ABS via sclerotherapy needle or heater probe catheter by the same senior endoscopist (E.O.). The vials of ABS® (patent number 2007-0-114485 were provided by Ankaferd Drug Inc., Istanbul, Turkey (one vial of 100 ml).

Periprocedural control of bleeding was attained in all instances and persisted for a few days except a fatal rebleeding after 16 hours in the patient with duodenal varix [Table 2].

**Figure 1:**
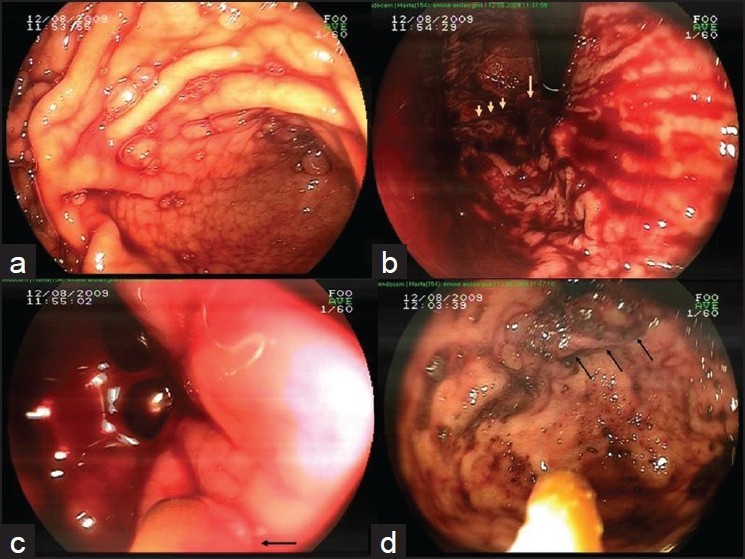
Case 2. Active bleeding (a) due to spurting gastric varix on retroflexion (b). ABS spraying via heater probe catheter in a random manner from distal esophagus (c), caused formation of ABS-induced plugs (arrows) and bleeding stopped (d)

**Figure 2:**
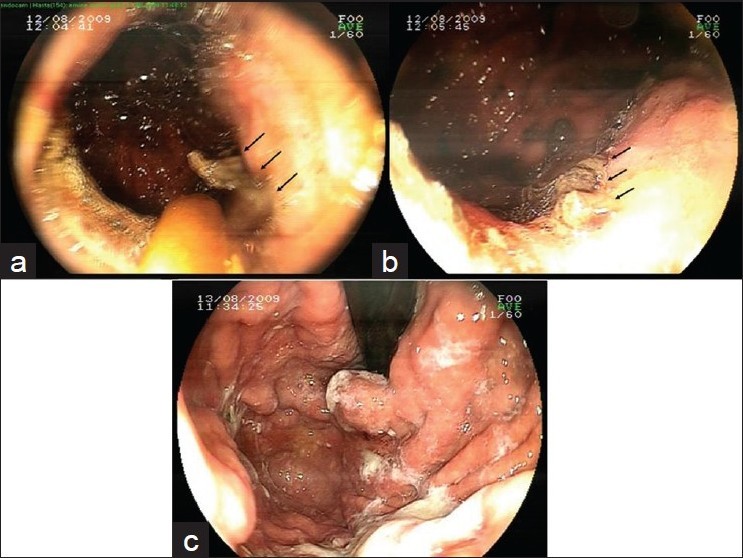
Case 2 (continued). Ankaferd Blood Stopper-induced coagulum are apparent on forward views at the proximal stomach (arrows) (a-b). Completely cleared stomach with gastric varix on the following day (c)

Endoscopic diagnoses were massively bleeding gastric varices in patients 1 to 3 [Figures 1 and 2]. They became hemodynamically unstable towards the end of the procedure. At that time, repeating the procedure via therapeutic endoscope with good suction capabilities or prokinetic agents to clear the visual field, Sangstaken-Blakemore tube or conservative measures were the options to bridge for a more definitive measure. We used 20 ml (for patient 1) and 15 ml (for patients 2 and 3) ABS solution that was sprayed in a random, untargeted fashion, with a heater probe catheter onto the bleeding areas beginning from the distal esophagus [Figure 1]. Thereafter, targeted application was tried as bleeding decreased. This produced greyish-yellow coagulum like a sludge throughout the stomach and bleeding stopped within minutes [Figure 2]. The cases were put on somatostatin and proper conservative therapy. In control endoscopy, patients 1 and 2 were made to undergo cyanoacrylate injection as a definitive therapy (1 ml Glubran^®^(GEM, Viareggio, Italy) plus 1.6 ml Lipiodol^®^(Guerbet Laboratories, Aulnay-sous-Bois, France). During the procedure on patient 2, massive bleeding occured after the second injection which decreased after further injections. However, massive hematemesis after 1 hour prompted us to perform the third endoscopy. ABS (25 ml) was reapplied and bleeding was stopped again. Patient 3 who had very large variceal columns, underwent a successful TIPSS and coil embolization at the seventh day of follow-up.

Patient 4 with alcoholic cirrhosis presented with relatively good hemodynamic features despite low hemoglobin level [Table 1]. In upper endoscopy, moderate fresh bleeding and fissuring on a large varix was detected at the distal duodenum. ABS application induced hemostasis for 6 hours during admission, however, the patient subsequently discharged himself against medical advice.

He came again the same night with re-bleeding, but died before any attempt could be made to stop the bleeding.

## DISCUSSION

Although many effective approaches to endoscopic hemostasis have evolved in recent decades, each has some restrictions. The endoscopist’s decision to use any one alone or in combination is determined largely by availability, personal preference and local expertise. Moreover, current endoscopic methods require identification of the bleeding focus. Excessive blood covering the examination field may cause diagnostic failure in 1.7 to 6.3% of non-variceal bleeding cases. This figure may be much higher in daily clinical practice especially with variceal bleeding, because many centers do not have therapeutic endoscopes and suitable aspiration devices while the endoscopist is usually forced to perform an early endoscopy. Our small case series showed that ABS may offer a useful adjunct in the therapeutic armamentarium of variceal bleeding cases due to the ease of application, non-toxicity and low cost. Neither any local adverse effect nor systemic toxicity was observed yet after the topical or even high-dose application of ABS.[4,5,10] However, intravariceal application of ABS should not be used due to its pro-coagulant action which may theoretically result in embolisation.

The major argument to the hemostatic effect of ABS may be spontaneous cessation of GI bleeding under conservative therapy. The bleeding was observed during endoscopy for a period of time to determine if it would stop spontaneously. Moreover, the durability of ABS hemostasis was defined upon the results of endoscopic observation during the procedure, subsequent clinical and laboratory follow-up, and control endoscopy [Table 2]. It is worth mentioning that we observed resolution of tachycardia during endoscopy upon ABS application in cases 2 and 3. The clinical rebleeding was assesed during hospitalization and after the procedure [Table 2]. So, the bleeding severity of our cases at presentation and the durability of the treatment which was 3 to 7 days for three cases (cases 1 to 3) and approximately 12 hours for one case (case 4), ensure the ABS induced immediate and short term hemostasis. Despite our encouraging preliminary experience, the bleeding could have stopped spontaneously and not necessarily due to intervention with ABS. Thus, further studies including a large number of patients are needed to clarify its short term effect and long term durability. Moreover, technical details of ABS application for GI bleeding are not settled yet. The size of injured point and bleeding vessel, the general hemostatic status and the underlying illness should be the main determinants of success similar to other measures. Limited experience[6–12] suggests, small doses of ABS stop bleeding from oozing lesions, within seconds. However, spurting lesions in our cases necessitated 7-25ml of ABS and bleeding stopped within a few minutes. We instilled ABS usually in a blind fashion due to severity of bleeding, but the exact localisation of bleeding point would lead to direct application of ABS onto the injured area with a smaller dose.

The basic mechanism of action for ABS is the formation of an encapsulated protein network that provides focal attachment points for very rapid vital erythrocyte aggregation like an “erythrocyte magnet”, which is known as the hemostatic “ABS-web”. Macroscopically, it causes a brownish or greyish-yellow deposit at the bleeding areas [Figures 1 and 2]. Recently, ultrastructural and morphological analysis of ABS induced coagulum was illustrated under the scanning electron microscopy.[9] ABS-induced protein network enriched with blood cells, particularly erythrocytes, covers the primary and secondary hemostatic system without disturbing individual coagulation factors or platelets.[3,9]

In conclusion, we observed the hemostatic effect of ABS in variceal bleeding, prompting us to consider that it may be used as a bridge to rescue therapy in variceal bleeding. We could not fully define its further advantages or disadvantages from this small observational case series. In spite of our promising data, these findings are preliminary and need to be confirmed in further studies.
